# Measurement model data of academic resilience for students in senior high school of middle seminary

**DOI:** 10.1016/j.dib.2020.106669

**Published:** 2020-12-23

**Authors:** Mimpin Sembiring, Danardana Murwani, Marthen Pali, Imanuel Hitipeuw

**Affiliations:** a Faculty of Psychological Education, State University of Malang, Indonesia; b Santo Bonaventura Pastoral High School, Medan, Indonesia; c Faculty of Economics, State University of Malang, Indonesia; d Faculty of Psychological Education, State University of Malang, Indonesia

**Keywords:** Student academic resilience, Secondary seminary, Validity, Reliability

## Abstract

This article aims to describe the academic resilience of secondary seminary students. Data were obtained from Garum Middle Seminary High School students, Blitar (East Java), Indonesia, in the 2019 academic year. Evidence for validity and reliability of the measurement was provided through confirmatory factor analysis. Previous research has used expert judges [1] to identify 100 items measuring academic resilience, encompassing four subscales (determination, endurance, adaptability, and recuperability). The current research used 28 of those items with the highest level of validity to create a 16-item measure of academic resilience.

## Specifications Table

**Table:** 

Subject	Psychology
Specific subject area	Educational Psychology, Academic Resilience
Type of data	Table and figure
How data were acquired	This data was obtained using a questionnaire to measure students academic resilience.
Data Format	Raw and Analyzed
Parameters for data collection	Participants were 113 high school seminary students from Blitar, East Java, Indonesia.
Description of data collection	Participants responded to 28 items of the academic resilience questionnaire, using a scale from 1 (strongly disagree) to 5 (strongly agree).
Data source location	Seminari High School St. Vincentius a Paulo Blitar Jl. Merdeka Timur 4–6 Garum, Blitar, East Java, Indonesia.
Data accessibility	The data files are available through Mendeley Data: http://dx.doi.org/10.17632/n93xpwdk6p.9

## Value of the Data

• The data are useful for measuring the level of academic resilience of high school students in secondary seminary, and finding possible factors that influence the level of resilience.
• The data are useful for providing input to policymakers, teachers, and especially seminary coaches to increase the academic resilience of their students.
• These data can be used to conduct longitudinal studies examining the development of academic resilience of high school students in middle seminary.

## 1 Data Description

This paper contains psychometric data for the measurement of academic resilience in high school seminary students. To our knowledge, this article is the first to measure the academic resilience of secondary seminary students. Academic resilience is the dynamic ability of students to succeed in studies despite experiencing many disturbances or pressures and problems [2,3]. In the current study, resilience was measured using the four dimensions proposed by Taormina [4]: determination, endurance, adaptability, and recuperability.

## 2 Experimental Design, Materials and Methods

**Fig. 1:**
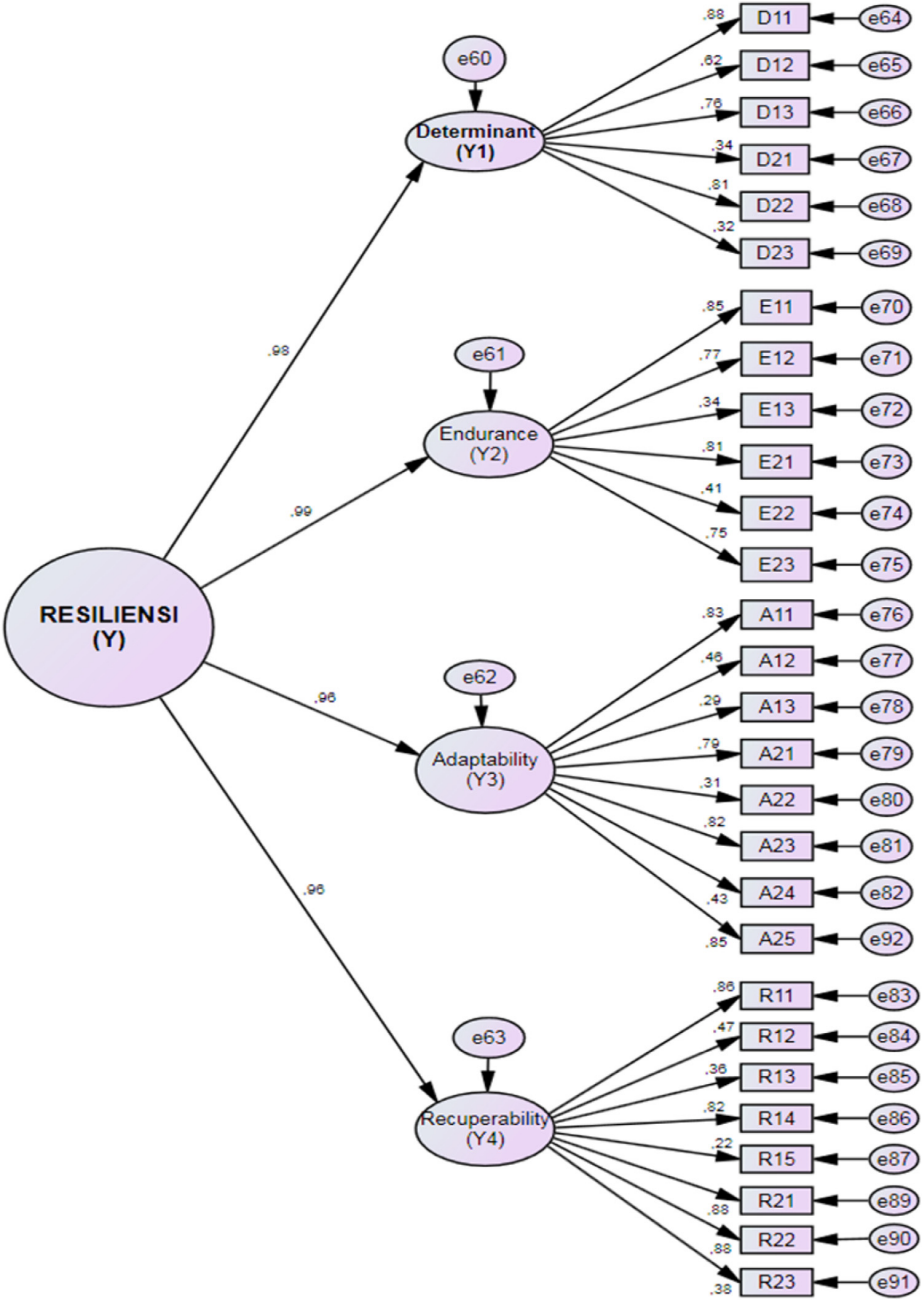
Confirmatory factor analysis results on 28 items.

**Fig. 2:**
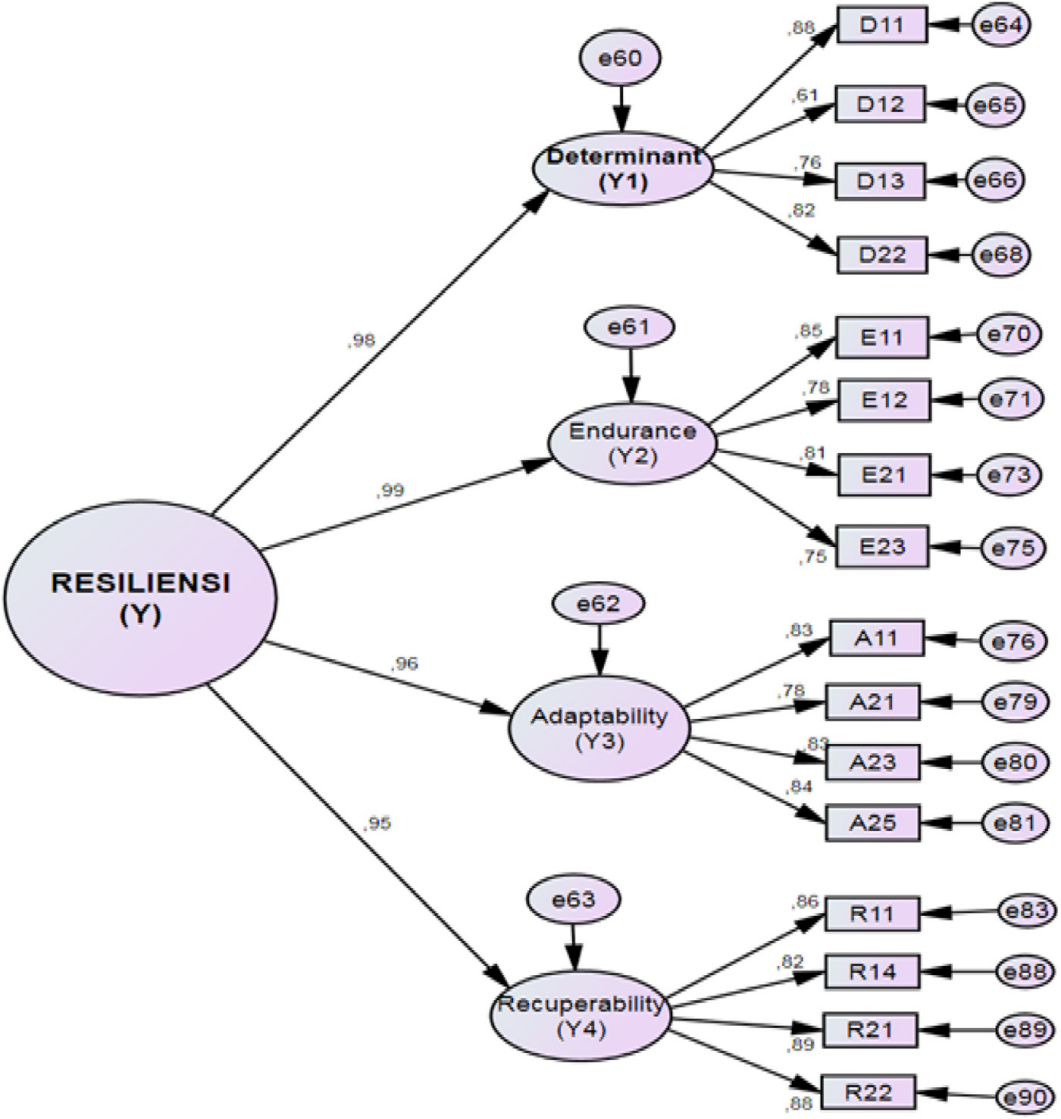
Confirmatory factor analysis results for 16 items.

Data collection was done by the researcher at the Garum Middle Seminary High School, Blitar, East Java, Indonesia. All students in this seminary are male. Their age range is 16–19 years. Respondents were 113 people, with 40 students in Class X, 42 students in Class XI, and 31 students in Class XII. This study was a non-experimental study using a questionnaire. The questionnaire contained 28 statement items, originally developed by Taormina which can be found in Table 1. Participants responded to each item using a five-point Likert scale ranging from 1 (strongly disagree) to 5 (strongly agree). Eight out of twenty eight items are un-favorable items, so when respondents chose option 5 on a scale (strongly agree), it means respondents’ resilience is lower. Responses in the data file have already been recoded as necessary (Fig. 1, Fig. 2).

**Table 1:** Items of measurement scale.

Dimension	No.	Code	Items
Determination	1	D11	I entered this seminary high school so I could become a priest
	2	D12	I never give up in the struggle to achieve goals
	3	D13	I would be grateful if I could step up in class every year
	4	D21	I entered this seminary high school so I could be accepted at the favorite university
	5	D22	I struggled hard so I could graduate from high school this seminary
	6	D23	I chose this middle school seminary because I was just a friend
Endurance	7	E11	I try to establish good relationships with all teachers
	8	E12	I maintain good relations with seniors
	9	E13	I find it hard to maintain friendships with classmates
	10	E21	I studied the subject matter over and over again that I had difficulty understanding
	11	E22	I pay extra attention to fields of study that I find difficult to understand
	12	E23	I find it hard to maintain concentration during the last lesson
Adaptability	13	A11	I can adjust to all the rules in this school
	14	A12	I feel bored with the monotonous schedule at this seminary high school
	15	A13	This school discipline trains my independence
	16	A21	I perform the evening worship whole heartedly
	17	A22	I get used to living with strict discipline
	18	A23	I carry out the cleaning duties with pleasure
	19	A24	I feel that seminary cleanliness is not my responsibility
	20	A25	I can ask senior brother for help with difficult subject matter
Recuperability	21	R11	I diligently exercise to maintain health
	22	R12	I fell ill from exhaustion washing and ironing clothes
	23	R13	My spirit recovered soon after recovering from this illness
	24	R14	My spirit rose again after serving the sentence for my offense
	25	R15	I was mentally devastated after being sentenced by the Board of Trustees
	26	R21	Teacher's stern admonition in academic guidance motivated me to be even better
	27	R22	I studied extra hard to improve my low grades
	28	R23	I study harder to catch up with some lessons

**Table 2:** CFA results.

		Partial Validity		
		(LF > 0,5=Valid)		
Latent Variable	Manifest Variable	Loading Factors	Note	Rank
Determinant (Y1)	D11	0.880	Valid	1
	D12	0.622	Valid	4
	D13	0.764	Valid	3
	D21	0.336	Invalid	5
	D22	0.808	Valid	2
	D23	0.317	Invalid	6

Endurance (Y2)	E11	0.849	Valid	1
	E12	0.769	Valid	3
	E13	0.341	Invalid	6
	E21	0.810	Valid	2
	E22	0.409	Invalid	5
	E23	0.754	Valid	4

Adaptability (Y3)	A11	0.832	Valid	2
	A12	0.455	Invalid	5
	A13	0.292	Invalid	8
	A21	0.787	Valid	4
	A22	0.311	Invalid	7
	A23	0.822	Valid	3
	A24	0.433	Invalid	6
	A25	0.848	Valid	1

Recuperability (Y4)	R11	0.858	Valid	3
	R12	0.468	Invalid	5
	R13	0.363	Invalid	7
	R14	0.824	Valid	4
	R15	0.223	Invalid	8
	R21	0.883	Valid	1
	R22	0.879	Valid	2
	R23	0.380	Invalid	6

Data analysis was performed using SPSS for descriptive statistics, and using AMOS to obtain Confirmatory Factor Analysis (CFA), Average Variance Extracted (AVE), and Composite Reliability (CR) values. Table 3 shows that the value of the loading factors of the 16 items ranged from 0.747 to 0886. The AVE value is between 0.782 and 0.896, and the CR value of each domain ranges from 0.855 to 0.921.

**Table 3:** Goodness of fit.

Goodness of fit Index	Results
Chi-Square	225.244
Probability	0.000
CMIN/DF	2.230
RMSEA	0.074
GFI	0.904
AGH	0.871
CFI	0.915

**Table 4:** Item, CFA, AVE, and CR.

Dimension	Code	Items	Loading Factors	AVE	CR
Determination	D11	I entered this seminary high school so I could become a priest	0.882	0.782	0.855
	D12	I never give up in the struggle to achieve goals	0.608		
	D13	I would be grateful if I could step up in class every year	0.763		
	D22	I struggled hard so I could graduate from high school this seminary	0.818		
Endurance	E11	I try to establish good relationships with all teachers	0.853	0.817	0.875
	E12	I maintain good relations with seniors	0.775		
	E21	I studied the subject matter over and over again that I had difficulty understanding	0.814		
	E23	I find it hard to maintain concentration during the last lesson	0.747		
Adaptability	A11	I can adjust to all the rules in this school	0.827	0.847	0.891
	A21	I perform the evening worship wholeheartedly	0.782		
	A23	I carry out the cleaning duties with pleasure	0.826		
	A25	I can ask senior brother for help with difficult subject matter	0.844		
Recoverability	R11	I diligently exercise to maintain health	0.856	0.896	0.921
	R14	My spirit rose again after serving the sentence for my offense.	0.824		
	R21	Teacher's stern admonition in academic guidance motivated me to be even better	0.886		
	R22	I studied extra hard to improve my low grades	0.883

According to Hair et al. [5], items with a loading factor of at least 0.5 are valid, suggesting that 16 of the items were valid, and 12 of the items are invalid. The results of the CFA suggest that the 16 valid items loaded onto four factors – determination (Y1; items D11, D12, D13, and D22), endurance dimensions (Y2; items E11, E12, E21, and E23); adaptability dimensions (Y3; items A11, A21, A23, A25), and recuperability (Y4; items R11, R14, R21, and R22). The goodness-of-fit coefficients for this model can be found in Table 4.

## Ethics Statement

The author states that all respondents, with their free will, agree to be included as the participants of this study by providing their answers to all of the questionnaires, without coercion from any parties. Their personal information is kept confidential.

## CRediT Authors Statement

**Mimpin Sembiring:** Conceptualization, Methodology, Writing-Preparation of original draft; **Danardana Murwani:** Software, Data Curation, Validation; **Marthen Pali:** Resources, Monitoring; **Imanuel Hitipeuw:** Reviewing and Editing, Project Administration

## Declaration of Competing Interest

The authors declare that they have no financial interests or personal relationships that could affect the work reported in this article.
